# Time trends in deaths before age 50 years in people with type 1 diabetes: a nationwide analysis from Scotland 2004–2017

**DOI:** 10.1007/s00125-020-05173-w

**Published:** 2020-05-26

**Authors:** Joseph E. O’Reilly, Luke A. K. Blackbourn, Thomas M. Caparrotta, Anita Jeyam, Brian Kennon, Graham P. Leese, Robert S. Lindsay, Rory J. McCrimmon, Stuart J. McGurnaghan, Paul M. McKeigue, John A. McKnight, John R. Petrie, Sam Philip, Naveed Sattar, Sarah H. Wild, Helen M. Colhoun, for the Scottish Diabetes Research Network Epidemiology Group

**Affiliations:** 1 MRC Institute of Genetics and Molecular Medicine, University of Edinburgh, Western General Hospital, Crewe Road, Edinburgh, EH4 2XUT UK; 2 Queen Elizabeth University Hospital, Glasgow, UK; 3 Ninewells Hospital, Dundee, UK; 4 Institute of Cardiovascular and Medical Sciences, University of Glasgow, Glasgow, UK; 5 Division of Molecular and Clinical Medicine, University of Dundee, Dundee, UK; 6 Usher Institute of Population Health Sciences and Informatics, Centre for Population Health Sciences, School of Molecular, Genetic and Population Health Sciences, University of Edinburgh, Edinburgh, UK; 7 Western General Hospital, NHS Lothian, Edinburgh, UK; 8 Grampian Diabetes Research Unit, Diabetes Centre, Aberdeen Royal Infirmary, Aberdeen, UK; 9 Public Health, NHS Fife, Kirkcaldy, UK

**Keywords:** DKA, Epidemiology, Mortality, Type 1 diabetes

## Abstract

**Aims/hypothesis:**

We aimed to examine whether crude mortality and mortality relative to the general population below 50 years of age have improved in recent years in those with type 1 diabetes.

**Methods:**

Individuals with type 1 diabetes aged below 50 and at least 1 year old at any time between 2004 and 2017 in Scotland were identified using the national register. Death data were obtained by linkage to Scottish national death registrations. Indirect age standardisation was used to calculate sex-specific standardised mortality ratios (SMRs). Poisson regression was used to test for calendar-time effects as incidence rate ratios (IRRs).

**Results:**

There were 1138 deaths in 251,143 person-years among 27,935 people with type 1 diabetes. There was a significant decline in mortality rate over time (IRR for calendar year 0.983 [95% CI 0.967, 0.998], *p* = 0.03), but the SMR remained approximately stable at 3.1 and 3.6 in men and 4.09 and 4.16 in women for 2004 and 2017, respectively. Diabetic ketoacidosis or coma (DKAoC) accounted for 22% of deaths and the rate did not decline significantly (IRR 0.975 [95% CI 0.94, 1.011], *p* = 0.168); 79.3% of DKAoC deaths occurred out of hospital. Circulatory diseases accounted for 27% of deaths and did decline significantly (IRR 0.946 [95% CI 0.914, 0.979], *p* = 0.002).

**Conclusions/interpretation:**

Absolute mortality has fallen, but the relative impact of type 1 diabetes on mortality below 50 years has not improved. There is scope to improve prevention of premature circulatory diseases and DKAoC and to develop more effective strategies for enabling people with type 1 diabetes to avoid clinically significant hyper- or hypoglycaemia.

**Figure:**
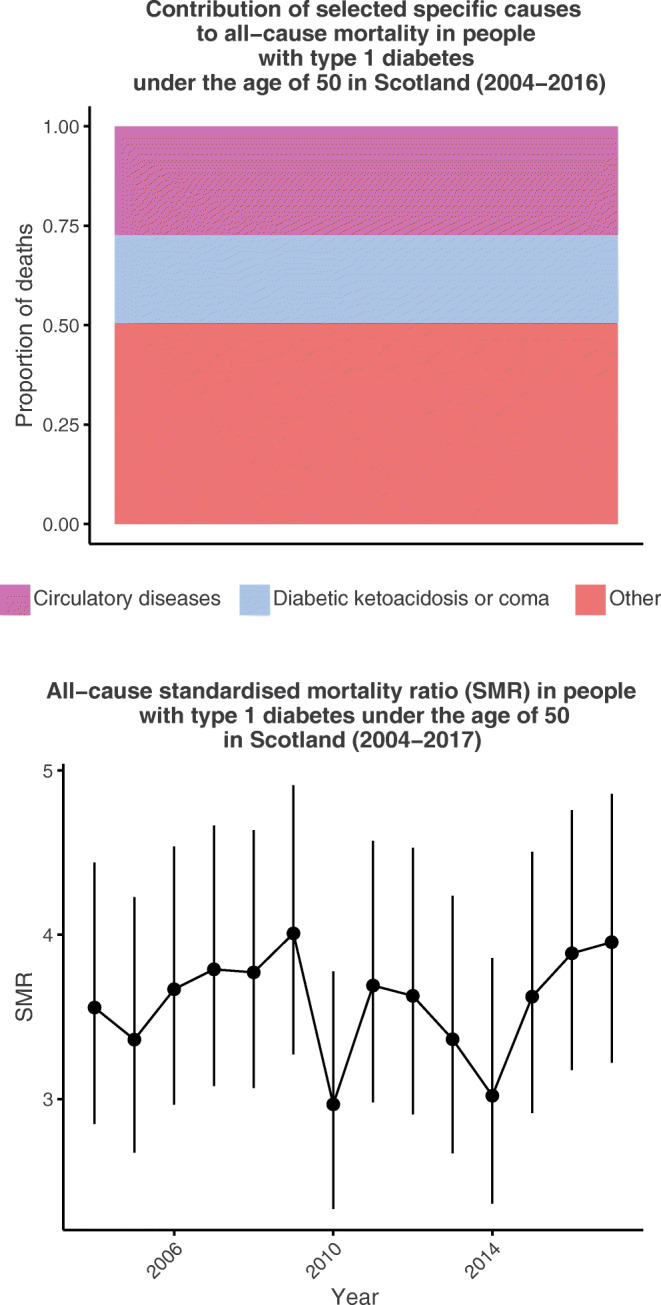
Graphical abstract

**Electronic supplementary material:**

The online version of this article (10.1007/s00125-020-05173-w) contains peer-reviewed but unedited supplementary material, which is available to authorised users.

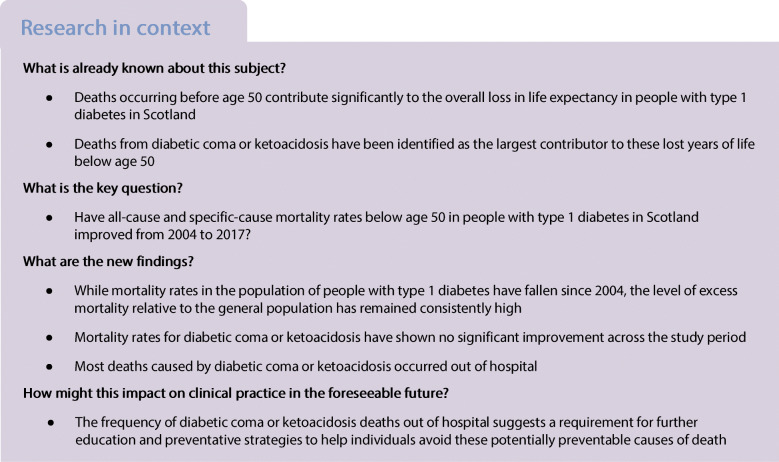



## Introduction

Type 1 diabetes mellitus is associated with excess mortality and elevated rates of mortality from many causes, with cardiovascular and renal disease contributing considerably to the excess mortality [1, 2]. There have been many advances in cardiovascular disease prevention in the past 15 years, including the widespread use of statin therapy in people both with and without diabetes [3]. There is evidence of declining cardiovascular mortality [4] and overall falls in mortality in type 1 diabetes that are faster than in the background population in Denmark (2002–2011) [5].

Deaths occurring below age 50 in people with type 1 diabetes, although fewer in number than in older populations, have a large impact on reduction in life expectancy. For example, we reported that 40–45% of the overall loss in life expectancy of 11 and 13 years in men and women with type 1 diabetes, respectively, was attributable to deaths occurring below age 50 [1]. Whether there have been any improvements in the prevention of these early deaths in type 1 diabetes in recent years is uncertain, as all recent studies have been very small or have had relatively short follow-up times, with the largest study including 3192 individuals [6–9]. Thus, the scope for further improvements in life expectancy by preventing such deaths is also unknown.

To address these questions, we examined whether death rates below age 50 years in type 1 diabetes have fallen since 2004 in a much larger dataset (*n* > 27,000) than previous studies. We further examined the pattern of deaths by cause and explored the extent to which deaths are occurring out of hospital as this has important implications for preventative strategies.

## Methods

### Dataset

Scottish Care Information - Diabetes (SCI-Diabetes) is the national disease management system of individuals diagnosed with any form of diabetes in Scotland and achieves over 99% coverage. As described previously [1], this register can be linked to other health record datasets including death data provided by the Information Services Division of National Health Service (NHS) Scotland and National Records for Scotland (NRS). We identified a cohort of all people with a clinical diagnosis of type 1 diabetes and who were aged below 50 years and at least 1 year old at any point during 2004–2017. Clinician-assigned diabetes type for each individual was accepted unless contradictory prescription history or age-at-diagnosis data were available (e.g. a clinician-assigned diagnosis of type 1 diabetes without any subsequent insulin prescription would not be accepted) [1]. Such individuals contributed data from 2004 or the date of their diagnosis with type 1 diabetes, whichever was later, up to date of death during the study period, attaining an age of 50 years, leaving the jurisdiction or the end of the study period. Individuals were considered to have left the jurisdiction if their electronic health record data showed a cessation of prescriptions or attendance of routine observations. Person-time below 1 year of age was not included in the cohort so as not to introduce immortal time bias.

The underlying cause of death was taken from the ICD10 code (http://apps.who.int/classifications/icd10/browse/2016/en) assigned on the Medical Certificate of Cause of Death (MCCD) issued following a death. We aggregated individual ICD10 codes into larger groups that defined high-level body systems or specific acute complications of diabetes. Assigning cause-specific mortality in diabetes has a number of challenges; for example, coding rules assign diabetes as the underlying cause of death where myocardial infarction is recorded on the MCCD as the underlying cause. Accordingly, when diabetes was the underlying cause of death, but a cardiovascular condition was an antecedent cause, we reassigned the underlying cause as circulatory disease. Similarly, we placed all deaths attributable to ‘diabetes mellitus with renal complications’ into the genitourinary system category, and all deaths attributable to ‘diabetes with peripheral circulatory complications’ into the circulatory system category. Furthermore, it should be noted that ICD10 coding of coma deaths does not differentiate between ketoacidotic or hypoglycaemic coma. Therefore, deaths due to diabetic ketoacidosis (DKA) or hypoglycaemic or unspecified coma were placed into a single category labelled ‘diabetic ketoacidosis or coma’ (DKAoC). The specific assignment of individual ICD10 codes to higher-level categories is detailed in electronic supplementary material (ESM) Table 1.

Through linkage with NRS data we were able to identify deaths for which a post-mortem examination had been performed. We were also able to identify which deaths had occurred in hospital by comparing an individual’s date of death with their date of discharge in the hospital admission data.

### Statistical methods

Crude mortality rates were calculated by calendar year across the entire study period and stratified by attained age band, sex and specific cause. Indirectly standardised mortality ratios (SMRs) were obtained by applying nationally reported overall and cause-specific mortality rates by age and sex for the general population to the type 1 diabetes population to estimate expected deaths, against which we compared observed deaths. Cause-specific comparisons were restricted to age <45 years only as this was the age band cut off used in the general population data. The area-based socioeconomic indicator the Scottish Index of Multiple Deprivation (SIMD) was used to assign the deprivation quintile to which the person with type 1 diabetes belonged at entry. For comparison, mortality rates stratified by SIMD quintile for the general population during the period 2008–2013 were available from NRS.

For presenting changes in rates over time among those with type 1 diabetes we first compared rates for the first and second halves of the study period, with each directly standardised to the type 1 diabetes age and sex structure in the middle year, i.e. 2011. In addition, Poisson regression models were used to estimate incidence rate ratios (IRRs) per calendar year, with 2004 as the baseline year, associated with sex, SIMD and calendar time, all adjusted by age and diabetes duration among those with type 1 diabetes. Poisson regression was also used to estimate IRRs for deaths by specific cause, adjusted for the covariates outlined above. To test for changes in the trends for all-cause and cause-specific mortality rates over time, we applied a joinpoint regression framework [10]. This method can identify the number and location of statistically significant changes in mortality rate trends over time.

## Results

### Total mortality

#### Absolute total mortality rates across the period 2004–2017

There were 27,935 people diagnosed with type 1 diabetes and aged <50 and >1 year at any point between 2004 and 2017. The mean age of the cohort across the study period was 30.56 years, and the cohort was 44% female. There were 1138 deaths. The total follow-up time was 251,143 person-years. The percentage of possible follow-up time that was not observed was 2.81%. Cohort characteristics at the start and end of the study period are presented in ESM Table 2 and the cohort age structure over time is presented in ESM Fig. 1. As shown in Table 1, the absolute mortality rate per 100,000 person-years across the study period was 453.13 (95% CI 427.55, 480); 500.2 in men (95% CI 464.65, 538.46) and 392.54 in women (95% CI 357.18, 431.4). Across the entire study period there were no deaths of individuals diagnosed with type 1 diabetes in the 1–9 age band. The overall SMR across the period was 3.42 (95% CI 3.23, 3.63). Although age-specific absolute mortality rates were mostly lower in females than males, SMRs at all age bands were higher for females than males (Table 1). The highest SMR was observed in females aged 20–29, where there were 73 deaths compared with 8–9 expected deaths.

**Table 1:** Deaths and excess deaths stratified by age band and sex across the entire study period

Age	Sex	N	Person-Years	Crude Rate^a^	Expected N	SMR (95% CI)
1–9	F	0	5156.11	0.00	0.58	–
1–9	M	0	5546.94	0.00	0.81	–
10–19	F	19	20,511.44	92.63	3.78	5.03 (3.21, 7.89)
10–19	M	28	22,754.83	123.06	7.50	3.73 (2.58, 5.41)
20–29	F	73	25,016.61	291.82	8.75	8.34 (6.63, 10.49)
20–29	M	83	32,577.34	254.78	30.55	2.72 (2.19, 3.37)
30–39	F	128	27,847.34	459.65	23.02	5.56 (4.68, 6.61)
30–39	M	220	37,280.00	590.13	65.03	3.38 (2.96, 3.86)
40–49	F	211	31,267.49	674.82	57.14	3.69 (3.23, 4.23)
40–49	M	376	43,187.42	870.62	135.51	2.77 (2.51, 3.07)
Total	M+F	1138	251,143.52	453.13	332.68	3.42 (3.23, 3.63)
Total	F	431	109,798.99	392.54	93.27	4.62 (4.20, 5.08)
Total	M	707	141,345.53	500.20	239.41	2.95 (2.74, 3.18)

^a^Crude rate is expressed per 100,000 person-years

F, female; M, male

#### Time trends in absolute total mortality and SMRs

**Fig. 1:**
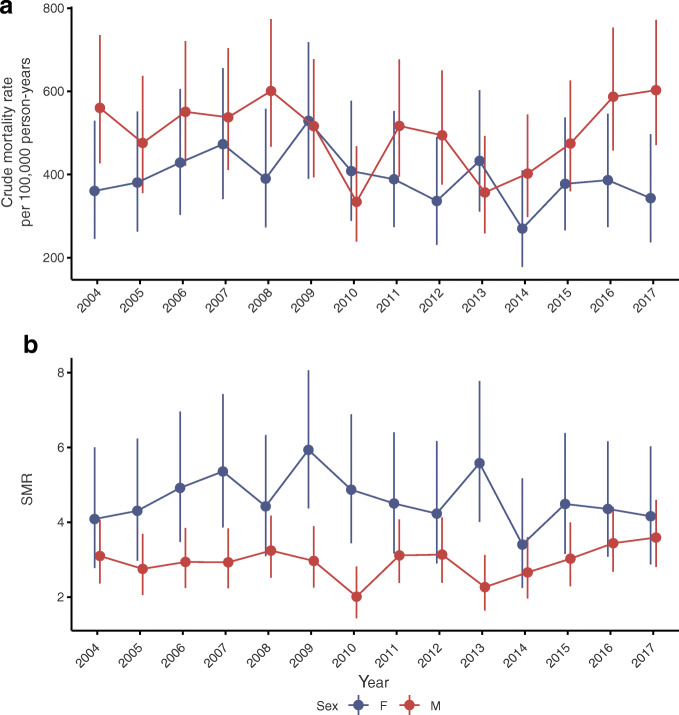
(**a**) Crude mortality rate and (**b**) SMR across the study period, stratified by sex. Points and bars for each year are slightly staggered to enhance visibility. Error bars show 95% CI. F, female; M, male

Figure 1 shows the crude mortality rates over time by sex, with no obvious trend discernible against the variation year to year. However, in the first half of the study period the total mortality rate (directly standardised to the 2011 age and sex distribution in those with type 1 diabetes) was 474.14 per 100,000 person-years (95% CI 468.58, 479.71) compared with 441.07 (95% CI 435.86, 446.28) in the second half. When calendar year was modelled as a categorical variable, a likelihood ratio test against the null model supported heterogeneity in yearly mortality rates (*p* = 0.002). Using Poisson regression to test for any linear effect of calendar time among those with type 1 diabetes, adjusted for age, sex and diabetes duration, there was a significant reduction in mortality with time (IRR per year 0.983 [95% CI 0.967, 0.998], *p* = 0.03). Joinpoint regression did not identify a significant shift in the trend for directly standardised mortality rate over time (*p* = 0.062). Sex-stratified analyses found broadly similar point estimates for calendar time for males (IRR 0.985 [95% CI 0.965, 1.004]) and females (IRR 0.979 [95% CI 0.954, 1.004]). Similar point estimates for calendar-time effect were observed in those below age 30 vs those aged 30 or older (IRR 0.964 [95% CI 0.930, 1.000]; IRR 0.988 [95% CI 0.971, 1.005]).

During this period, mortality rates in the general population aged 1–49 years in Scotland also declined to a broadly similar extent (IRR for the effect of calendar time 0.987 [95% CI 0.985, 0.989], *p* < 0.001). Accordingly, there was no improvement in the SMR associated with diabetes (Fig. 1), going from 3.10 (95% CI 2.36, 4.07) to 3.60 (95% CI 2.81, 4.60) in males and 4.09 (95% CI 2.78, 6.01) to 4.16 (95% CI 2.88, 6.03) in females in 2004–2017. Similarly, joinpoint regression identified no significant shift in the SMR over time for males (*p* = 0.086) or females (*p* = 0.19).

#### The association of social deprivation with total mortality

We have previously reported large socioeconomic differences in achievement of glucose control in type 1 diabetes in our population [11]. Accordingly, among those with type 1 diabetes we explored the association of total mortality with social deprivation, adjusted for age, sex and diabetes duration. As age-, sex- and SIMD-specific mortality rates were available for the general population, we were also able to compute SIMD quintile-specific SMRs.

**Fig. 2:**
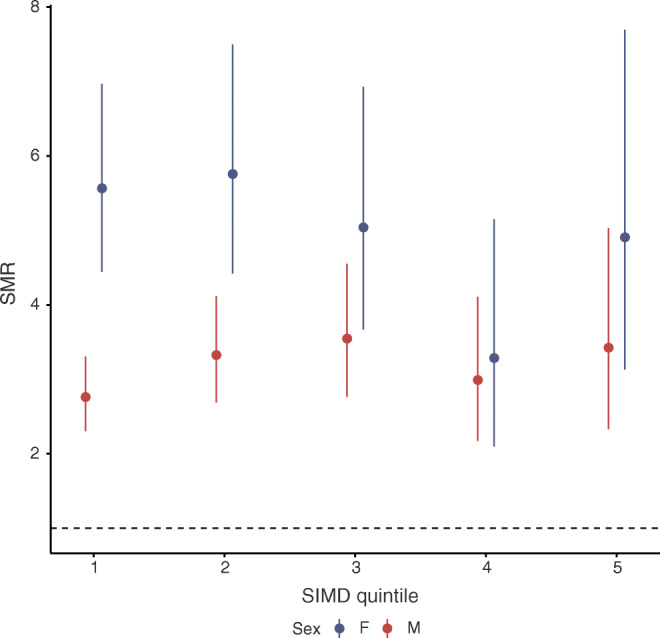
SMR by SIMD quintile and sex (2008–2013 only). The horizontal dotted line marks an SMR of 1. Error bars show 95% CI. F, female; M, male

Among those with type 1 diabetes, mortality varied strongly with SIMD quintile, with the IRR for the fifth living in the most-deprived vs the fifth living in the least-deprived quintile being 4.89 (95% CI 3.77, 6.43). IRRs for each SIMD quintile relative to the least-deprived quintile are presented in ESM Fig. 2. The effect appeared linear as the IRR for SIMD as a continuous term was 1.47 (95% CI 1.40, 1.54) with a *p* value of <0.001, so that risk escalated with each SIMD group. There was no evidence of any change in SIMD effects over time (SIMD×time interaction term in the Poisson model *p* values >0.188 for all quintiles). However, the SMR associated with diabetes compared with the background population was similar across SIMD quintiles (Fig. 2). Thus, while there are strong associations of deprivation with total mortality among those with type 1 diabetes, these are of a similar magnitude to the association of SIMD with mortality in the background population and have not changed over this period.

### Cause-specific mortality

#### Cause of death by age

Due to a lack of underlying cause for approximately 30 deaths that occurred in 2017 for which post-mortem results are pending, we excluded all data from this year when performing calculations and tabulations for cause-specific mortality.

Overall, 57% of deaths (*n* = 654: 81% of deaths out of hospital and 26% of deaths in hospital) had a post-mortem examination. Altogether, there were 188 deaths for which DKA was the underlying cause and one with hypoglycaemia as the underlying cause. There were 48 deaths that had the ICD10 code for ‘diabetes with coma’ as the underlying cause, of which zero had hypoglycaemia as a secondary cause and zero had DKA as a secondary cause. So, for 48 deaths it is unclear whether the coma was due to DKA or hypoglycaemia. We therefore used a combined term of ‘DKAoC’ to encompass all deaths due to either DKA or hypoglycaemia.

Of the 1047 deaths in the study period prior to 2017, the most common were circulatory system deaths (27%), followed by deaths due to DKAoC (22%) and external causes of mortality (9%). As shown in Table 2, the absolute rate of each cause rises with age, but most steeply so for circulatory disease and neoplasm deaths.

**Table 2:** Mortality and excess mortality stratified by specific cause and age band 2004–2016

Cause of death	Age	N (*N* < 45)	Crude rate (95% CI)^a^	SMR (95% CI)^b^	Proportion with post-mortem	Proportion in hospital
Certain infectious and parasitic diseases	15–24	–	1.86 (0.26, 13.2)	4.67 (0.66, 33.16)	0.00	1.00
Certain infectious and parasitic diseases	25–34	–	5.01 (1.62, 15.54)	4.39 (1.41, 13.6)	0.67	0.67
Certain infectious and parasitic diseases	35–44	–	18.09 (10.5, 31.15)	6.01 (3.49, 10.36)	0.23	0.85
Certain infectious and parasitic diseases	45–49	–	10.84 (4.07, 28.87)	–	0.00	1.00
Certain infectious and parasitic diseases	Total	21 (17)	8.36 (5.45, 12.82)	5.37 (3.34, 8.64)	0.24	0.86
DM other	15–24	–	14.88 (7.44, 29.75)	–	0.88	0.12
DM other	25–34	–	28.4 (17.65, 45.68)	–	0.76	0.29
DM other	35–44	–	50.09 (36.13, 69.45)	–	0.58	0.28
DM other	45–49	–	37.93 (22.46, 64.04)	–	0.57	0.43
DM other^c^	Total	75 (61)	29.86 (23.82, 37.45)	–	0.65	0.29
DM with DKAoC	10–14	–	16.64 (5.37, 51.6)	–	0.33	1.00
DM with DKAoC	15–24	–	87.4 (65.67, 116.33)	–	0.87	0.30
DM with DKAoC	25–34	–	101.9 (79.29, 130.97)	–	0.87	0.13
DM with DKAoC	35–44	–	121.06 (98.12, 149.37)	–	0.86	0.20
DM with DKAoC	45–49	–	94.82 (68.08, 132.07)	–	0.86	0.17
DM with DKAoC	Total	233 (198)	92.78 (81.6, 105.49)	–	0.86	0.21
Diseases of the circulatory system	15–24	–	11.16 (5.01, 24.84)	5.98 (2.69, 13.31)	0.67	0.83
Diseases of the circulatory system	25–34	–	58.47 (41.98, 81.43)	8.84 (6.34, 12.31)	0.66	0.49
Diseases of the circulatory system	35–44	–	197.59 (167.62, 232.92)	6.76 (5.73, 7.96)	0.47	0.57
Diseases of the circulatory system	45–49	–	276.34 (227.6, 335.53)	–	0.41	0.43
Diseases of the circulatory system	Total	285 (183)	113.48 (101.04, 127.45)	7 (6.06, 8.1)	0.48	0.52
Diseases of the digestive system	15–24	–	1.86 (0.26, 13.2)	3.04 (0.43, 21.58)	1.00	1.00
Diseases of the digestive system	25–34	–	10.02 (4.5, 22.31)	1.99 (0.89, 4.43)	0.50	0.50
Diseases of the digestive system	35–44	–	40.35 (28.04, 58.07)	1.94 (1.35, 2.79)	0.52	0.45
Diseases of the digestive system	45–49	–	46.06 (28.63, 74.09)	–	0.24	0.88
Diseases of the digestive system	Total	53 (36)	21.1 (16.12, 27.62)	1.96 (1.41, 2.72)	0.43	0.60
Diseases of the genitourinary system	25–34	–	20.05 (11.38, 35.3)	55.07 (31.28, 96.98)	0.25	0.42
Diseases of the genitourinary system	35–44	–	59.83 (44.37, 80.68)	68.59 (50.87, 92.49)	0.21	0.79
Diseases of the genitourinary system	45–49	–	40.64 (24.5, 67.41)	–	0.13	0.80
Diseases of the genitourinary system	Total	70 (55)	27.87 (22.05, 35.23)	61.44 (47.17, 80.02)	0.20	0.73
Diseases of the nervous system	10–14	–	5.55 (0.78, 39.38)	4.16 (0.59, 29.53)	0.00	1.00
Diseases of the nervous system	15–24	–	7.44 (2.79, 19.82)	2.5 (0.94, 6.65)	0.25	1.00
Diseases of the nervous system	25–34	–	11.69 (5.57, 24.53)	3.52 (1.68, 7.38)	0.57	0.57
Diseases of the nervous system	35–44	–	15.31 (8.48, 27.64)	2.77 (1.53, 5)	0.18	0.73
Diseases of the nervous system	45–49	–	18.96 (9.04, 39.78)	–	0.29	1.00
Diseases of the nervous system	Total	30 (23)	11.95 (8.35, 17.08)	2.89 (1.92, 4.35)	0.30	0.80
Diseases of the respiratory system	15–24	–	3.72 (0.93, 14.87)	3.64 (0.91, 14.56)	0.50	0.50
Diseases of the respiratory system	25–34	–	3.34 (0.84, 13.36)	1.8 (0.45, 7.19)	0.00	1.00
Diseases of the respiratory system	35–44	–	13.91 (7.49, 25.86)	2.4 (1.29, 4.46)	0.60	0.70
Diseases of the respiratory system	45–49	–	32.51 (18.46, 57.25)	–	0.17	0.67
Diseases of the respiratory system	Total	26 (14)	10.35 (7.05, 15.21)	2.31 (1.37, 3.9)	0.35	0.69
External causes of mortality	10–14	–	5.55 (0.78, 39.38)	2.09 (0.29, 14.82)	1.00	1.00
External causes of mortality	15–24	–	27.89 (16.82, 46.27)	1.01 (0.61, 1.68)	0.93	0.27
External causes of mortality	25–34	–	50.12 (35.04, 71.68)	1.14 (0.8, 1.64)	0.97	0.13
External causes of mortality	35–44	–	50.09 (36.13, 69.45)	0.93 (0.67, 1.29)	0.86	0.19
External causes of mortality	45–49	–	32.51 (18.46, 57.25)	–	0.83	0.25
External causes of mortality^d^	Total	94 (82)	37.43 (30.58, 45.81)	1.02 (0.82, 1.27)	0.90	0.20
Mental and behavioural disorders	15–24	–	5.58 (1.8, 17.3)	1.35 (0.44, 4.19)	1.00	0.33
Mental and behavioural disorders	25–34	–	15.03 (7.82, 28.9)	1.23 (0.64, 2.36)	1.00	0.00
Mental and behavioural disorders	35–44	–	23.66 (14.71, 38.05)	1.52 (0.95, 2.45)	0.82	0.12
Mental and behavioural disorders	45–49	–	21.67 (10.84, 43.34)	–	0.50	0.50
Mental and behavioural disorders	Total	37 (29)	14.73 (10.67, 20.33)	1.4 (0.97, 2.01)	0.81	0.19
Neoplasms	15–24	–	3.72 (0.93, 14.87)	1.07 (0.27, 4.28)	0.50	0.50
Neoplasms	25–34	–	16.71 (8.99, 31.05)	1.84 (0.99, 3.41)	0.00	0.70
Neoplasms	35–44	–	36.18 (24.63, 53.14)	1.17 (0.79, 1.71)	0.04	0.73
Neoplasms	45–49	–	113.79 (84.09, 153.97)	–	0.02	0.67
Neoplasms	Total	80 (38)	31.85 (25.59, 39.66)	1.26 (0.91, 1.72)	0.04	0.69
Other	15–24	–	11.16 (5.01, 24.84)	–	0.50	0.50
Other	25–34	–	23.39 (13.85, 39.49)	–	0.71	0.43
Other	35–44	–	25.05 (15.78, 39.75)	–	0.78	0.28
Other	45–49	–	13.55 (5.64, 32.55)	–	1.00	0.60
Other^e^	Total	43 (38)	17.12 (12.7, 23.09)	–	0.74	0.40

^a^Crude mortality rate is expressed per 100,000 person-years

^b^SMR calculations are restricted to individuals less than 45 years old due to restricted availability of data in the general population

^c^‘DM other’ consists of ICD10 codes for any form of diabetes with ‘unspecified complications’, ‘no complications’ or ‘multiple complications’

^d^‘External causes of mortality’ consists of ICD10 codes V01–Y98

^e^‘Other’ consists of ICD10 codes D50–89, L00–99, M00–99, O00–99, P00–99, R99 and Q00–99

DM, type 1 diabetes mellitus

**Fig. 3:**
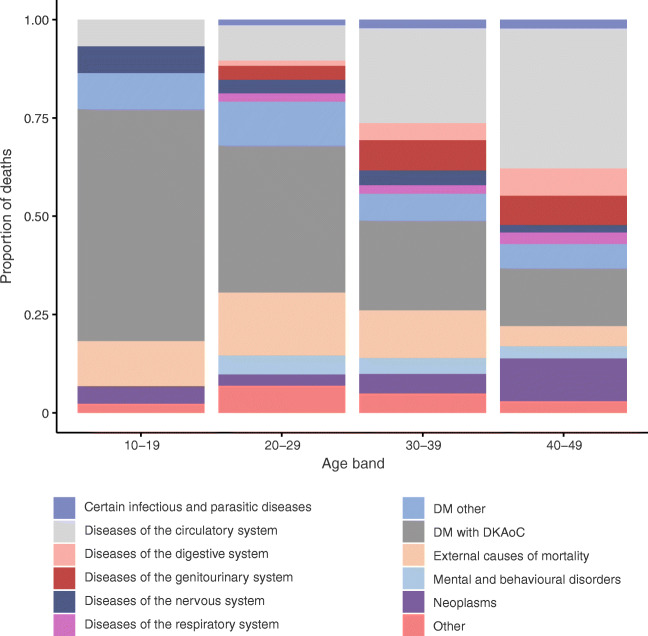
Underlying cause of death by age band. DM, type 1 diabetes mellitus

As shown in Fig. 3, below 30 years of age DKAoC deaths account for almost half the deaths (43%), whereas above this age they account for 18%. Of note is that 79% of DKAoC deaths occurred out of hospital. As with total mortality, DKAoC death rates varied by SIMD, with IRR for the most- vs least-deprived quintile 11.324 (95% CI 5.398, 29.074; *p*< 0.001); there were seven DKAoC deaths in the least-deprived quintile compared with 88 in the most-deprived quintile.

#### Cause-specific SMRs

**Fig. 4:**
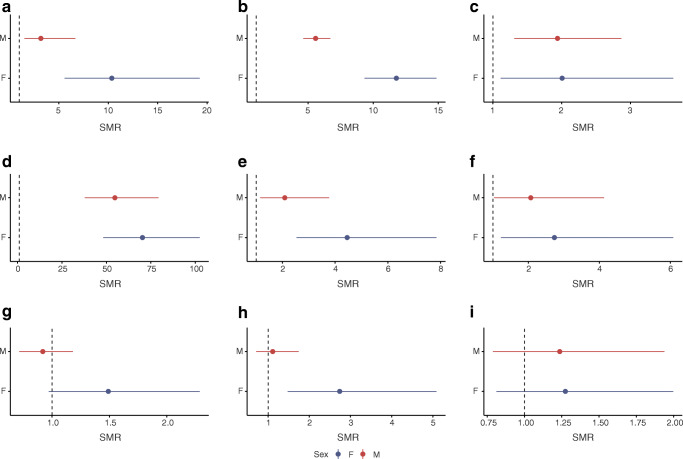
SMR stratified by specific cause and sex. Vertical dotted lines mark an SMR of 1. Error bars show 95% CI. Calculation of SMR was restricted to individuals aged <45 and to the period 2004–2016 owing to the restricted availability of data. (**a**) Certain infectious and parasitic diseases, (**b**) diseases of the circulatory system, (**c**) diseases of the digestive system, (**d**) diseases of the genitourinary system, (**e**) diseases of the nervous system, (**f**) diseases of the respiratory system, (**g**) external causes of mortality, (**h**) mental and behavioural disorders, (**i**) neoplasms. F, female; M, male

For causes of death other than those coded as diabetes, the age-standardised cause-specific SMRs are shown in Table 2 and Fig. 4. Cause-specific SMRs by age band are presented in ESM Fig. 3. There was a significant elevation in deaths, and not just circulatory and renal system deaths, as expected, but also infectious deaths and digestive, nervous and respiratory system deaths, as well as mental and behavioural deaths in women. Digestive system deaths mostly comprised alcoholic liver disease deaths (42%). Genitourinary deaths mostly comprised renal deaths (89%). For diseases of the nervous system the most frequent cause was epilepsy (37%). Diseases of the respiratory system mostly comprised pneumonia (38%). Mental and behavioural deaths mostly comprised alcohol or opioid dependence deaths (70%). The most common neoplasms were breast (16%) and lung (16%). External causes mostly comprised intentional and accidental poisoning (62%). The SMRs were larger in females than males for most cause categories.

#### Cause-specific rates over time

We examined time trends in the two most common causes of death. In the first half of the study period the rate of deaths caused by DKAoC per 100,000 person-years (directly standardised to the 2011 age distribution in those with type 1 diabetes) was 104.65 (95% CI 102.04, 107.26), compared with 96.39 (95% CI 93.32, 99.45) in the second half. There was minimal support for heterogeneity in the rate of DKAoC mortality across the study period (*p* = 0.12). Using Poisson regression adjusted for age, diabetes duration and sex to test for a linear time trend for deaths caused by DKAoC showed no significant downward trend (IRR per year 0.975 [95% CI 0.94, 1.011], *p* = 0.168). When the same Poisson regression model was applied with the outcome variable redefined to consist only of deaths with a definite underlying cause of DKA, then there was still no evidence of a significant downward trend over time (IRR per year 1.007 [95% CI 0.966, 1.049], *p* = 0.74). Similarly, joinpoint regression did not identify a significant shift in the trend of the rate of DKAoC mortality over time (*p* = 0.33). There were too few definite hypoglycaemia deaths to analyse separately.

In the first half of the study period the rate of deaths caused by diseases of the circulatory system per 100,000 person-years (directly standardised to the 2011 age distribution in those with type 1 diabetes) was 131.47 (95% CI 128.53, 134.41), compared with 116.51 (95% CI 113.13, 119.89) in the second half. Of the individuals with type 1 diabetes and a cause of death attributable to diseases of the circulatory system, 55% had a previous hospitalisation for either hypertensive disease or ischaemic heart disease at any point in time, 86% had ever received a prescription for any cardiovascular drug and 70% had ever received a prescription for any statin. SMRs for diseases of the circulatory system were 9.78 (95% CI 6.31, 15.16) in 2004 and 6.98 (95% CI 3.97, 12.3) in 2016. Using Poisson regression adjusted for age, diabetes duration and sex to test for a linear time trend for deaths caused by diseases of the circulatory system resulted in an IRR per year of 0.946 (95% CI 0.914, 0.979; *p* = 0.002); in the general population the age band- and sex-adjusted effect of calendar time on circulatory disease mortality was 0.970 (95% CI 0.961, 0.978; *p* < 0.001). Joinpoint regression did not identify a significant shift in the trend of the rate of circulatory disease mortality over time (*p* = 0.24).

## Discussion

### Key findings

This is one of the largest investigations of deaths under age 50 years in type 1 diabetes that has been conducted in recent years. We have deliberately focused on this age range because analyses focusing on the total population are primarily determined by circulatory system and cancer deaths at older ages, and patterns at younger ages are therefore often obscured. Yet deaths under age 50 account for half of all the total loss in life expectancy associated with type 1 diabetes [1], and the strategies needed to tackle this are somewhat different from those for reducing death rates at older ages. The key findings from our study are that between 2004 and 2017 there was an improvement in the absolute mortality rate under age 50 years, but that relative mortality showed no sign of improvement, with rates remaining between three- and fourfold higher among people with type 1 diabetes than the general population. SMRs were markedly higher in females than males. There was no clear evidence that the rate of DKAoC deaths fell, and the absolute rate remains high, with approximately 80 deaths per 100,000 population per year. Most of these deaths occur out of hospital. Rates of circulatory deaths, the most common cause of death, have fallen in this period, but not significantly more so than reductions in deaths from this cause in the background population. Death rates are elevated not just for the causes commonly recognised as diabetes complications (diabetes specific, circulatory and renal) but also for infectious diseases and diseases of the respiratory, digestive and nervous systems, demonstrating the impact of type 1 diabetes across all body systems. An ongoing large socioeconomic differential in death rates among people with type 1 diabetes has persisted across the period. For overall mortality rates these are of similar magnitude as socioeconomic differentials in the background population. However, for DKAoC deaths there was a disturbing 11-fold difference in the rates in the most- vs least-deprived quintiles of the population.

### Comparison with past literature

High SMRs at young ages in diabetes have been reported in other countries, but changes in absolute and relative mortality rates compared with the background population over time vary between studies. Studies in Australia (2000–2011), Finland (1970–1999) and Denmark (2002–2011) identified reductions in both absolute and relative mortality rates, whereas analyses in Sweden (1998–2011), Wales and Northern Ireland (1989–2012) found no improvement in relative mortality [2, 5–7, 12–14].

The greater relative effect of diabetes on CVD, renal disease, neoplasms, and accident and suicide in females than in males has been reported elsewhere [15]; here, we show that this greater effect of diabetes in females extends to other body systems, including respiratory disease and infectious disease deaths. We note poorer glycaemic control in females than males in our population [11], which is an observation made in other national populations of people with type 1 diabetes [16]. It has long been debated whether this reflects a biological or behavioural phenomenon [17], and we are not aware of any interventions that have been shown to alter it. Clearly, such exploratory and intervention studies are needed if we are to reduce this disparity in diabetes effect by sex.

### Strengths and limitations

Strengths of our work are the large size of the dataset enabling precise estimates of rates and effects. The limitations of our study mostly pertain to generalisability since our analysis has only been carried out within one country. Another limitation is that routine datasets have limitations in how extensively they can explore specific circumstances leading to death. Such detailed individual scrutiny of deaths, as is done, for example, in a confidential inquiry methodology, may be the most useful for informing preventative policies for DKA and coma deaths.

### Policy implications and conclusions

The lack of improvement in relative mortality and the persistent socioeconomic differential in this nationwide analysis emphasise the profound need to improve the lives of those with type 1 diabetes and to ensure that advances in care reach all sectors of society. In Scotland in the past decade or so there have been regularly renewed strategic plans to improve diabetes management in our national health system, including more widespread psychological support, improved access to structured education and widening of access to pumps, continuous glucose monitoring and flash glucose monitors. Yet, despite this, these theoretically preventable early deaths continue to occur. There has been no significant improvement in the rate of DKA and coma deaths and clearly more work needs to be done to achieve this. Our analysis shows that these deaths are not occurring through poor hospital management or poor inpatient care of those with glycaemic crises—most of the deaths occur out of hospital. This implies a need for greater education and preventative strategies targeted at people with diabetes, their peers and their families. Currently, all people with type 1 diabetes in Scotland receive education on insulin management during sickness and are provided with meters for measuring ketones. In addition, education is provided on the avoidance of hypoglycaemia as well as the impact of recreational drug use and alcohol. A previous analysis from one health board found that over 50% of DKA admissions are from individuals who have experienced two or more DKA events, and such individuals have a high risk of subsequent mortality [18]. This suggests that more intensive management of individuals after DKA is required. Of particular concern is the large socioeconomic differential in these deaths. It will be critically important to ensure that new strategies for use of pumps and blood glucose-monitoring devices reduce rather than widen this differential. It is also essential that pump therapy is introduced safely as these devices have been shown to increase the risk of acute metabolic complications in individuals with lower socioeconomic status [19]. With regard to the ongoing excess of circulatory deaths, the high rate of prior hospitalisation and prescriptions for hypertensive and ischaemic heart conditions suggests that the majority of these deaths are occurring in individuals with established circulatory disease rather than sudden deaths in people with no prior history. Our further work is focusing on building prediction models of those most at risk so that preventative strategies can be tailored to those at risk. Although the relative mortality rates for circulatory diseases are high, the absolute mortality rates are not, e.g. <5% 5 year mortality risk in the 45–49 age group (Table 2). In addition to targeted preventative strategies, population-level improvements in smoking cessation, glycaemic control, and blood pressure and lipid management are needed to drive this rate down further. The occurrence of renal disease deaths at a young age against a backdrop of national policies on annual albumin/creatinine ratio and eGFR checks and risk factor monitoring is also disturbing. We have learned a great deal about prevention of diabetes complications in recent decades, but many people with type 1 diabetes continue to experience a profound impact on health. There is no room for complacency. We need further initiatives to predict, identify and provide support to those most at risk of premature mortality from type 1 diabetes.

## Electronic supplementary material

ESM (PDF 118 kb)

## Data Availability

We do not have governance permissions to share individual-level data on which these analyses were conducted. However, for any genuine requests to audit the validity of the analyses, the verifiable research pipeline that we operate allows researchers to make a request to the corresponding author to view the analyses being run and the resulting tabulations, summary statistics and parameter estimates.

## Abbreviations

DKA: Diabetic ketoacidosis
DKAoC: Diabetic ketoacidosis or coma
IRR: Incidence rate ratio
MCCD: Medical Certificate of Cause of Death
NRS: National Records for Scotland
SIMD: Scottish Index of Multiple Deprivation
SMR: Standardised mortality ratio
