# Supplementary material for: Time trends in deaths before age 50 years in people with type 1 diabetes: a nationwide analysis from Scotland 2004–2017
Source: Diabetologia. 2020 May 26;63(8):1626–36. doi: 10.1007/s00125-020-05173-w (PMC7351819; doi:10.1007/s00125-020-05173-w)
Supplement: Supplementary file 1 — (PDF 118 kb) [file 125_2020_5173_MOESM1_ESM.pdf]

Time trends in deaths before age 50 years in people with type 1 diabetes: a nationwide analysis from Scotland 2004-2017. Electronic Supplementary Materials

Table 1: ICD10 groupings employed in analyses

Category	ICD10 Codes
Certain Infectious and Parasitic Diseases	A00-B99
Neoplasms	C00-D48
Mental and Behavioural Disorders	F00-99
Diseases of the Nervous System	G00-H95
Diseases of the Circulatory System	I00-99, E10.5, E11.5, E13.5, E14.5
Diseases of the Respiratory System	J00-99
Diseases of the Digestive System	K00-93
Diseases of the Genitourinary System	N00-99, E10.2, E11.2, E13.2, E14.2
External Causes of Mortality	V01-Y98
DM With DKA Or Coma	E10.0, E11.0, E13.0, E14.0, E10.1, E11.1, E13.1, E14.1, E15
DM Other	E10.6, E11.6, E13.6, E14.6, E10.7, E11.7, E13.7, E14.7, E10.8, E11.8, E13.8, E14.8, E10.9, E11.9, E13.9, E14.9
Other	All Other ICD10 Codes

Table 2: Cohort characteristics at beginning and end of study period

Y	Mean Age	% Female	Mean Diabetes Duration (Years)	N
2004	30.48	43.55	14.51	16911
2017	30.44	43.72	16.27	18886

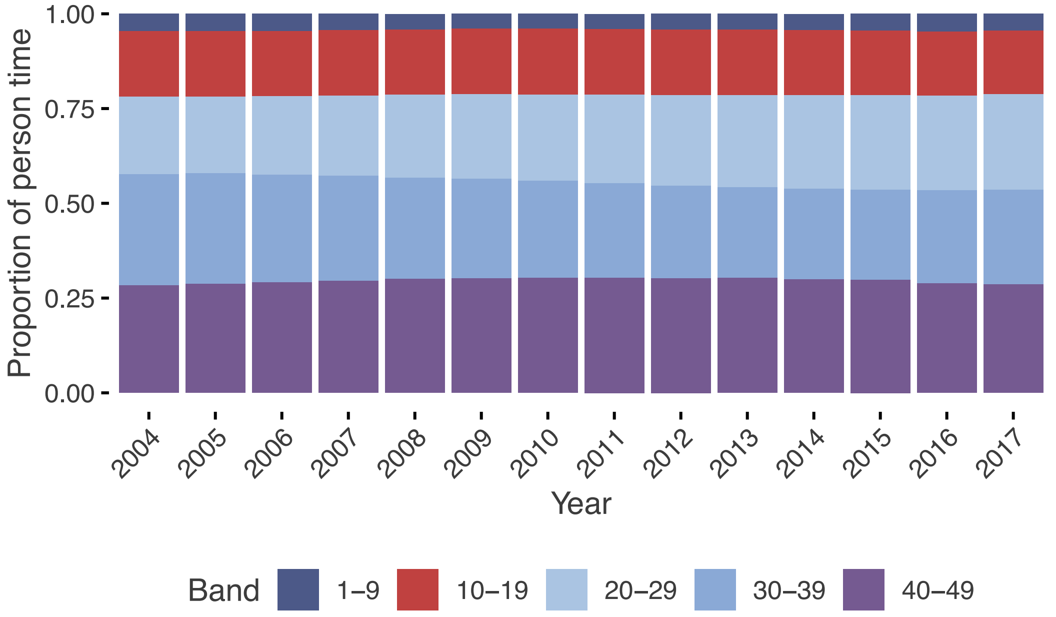

ESM figure 1. Cohort age band structure through the study period.

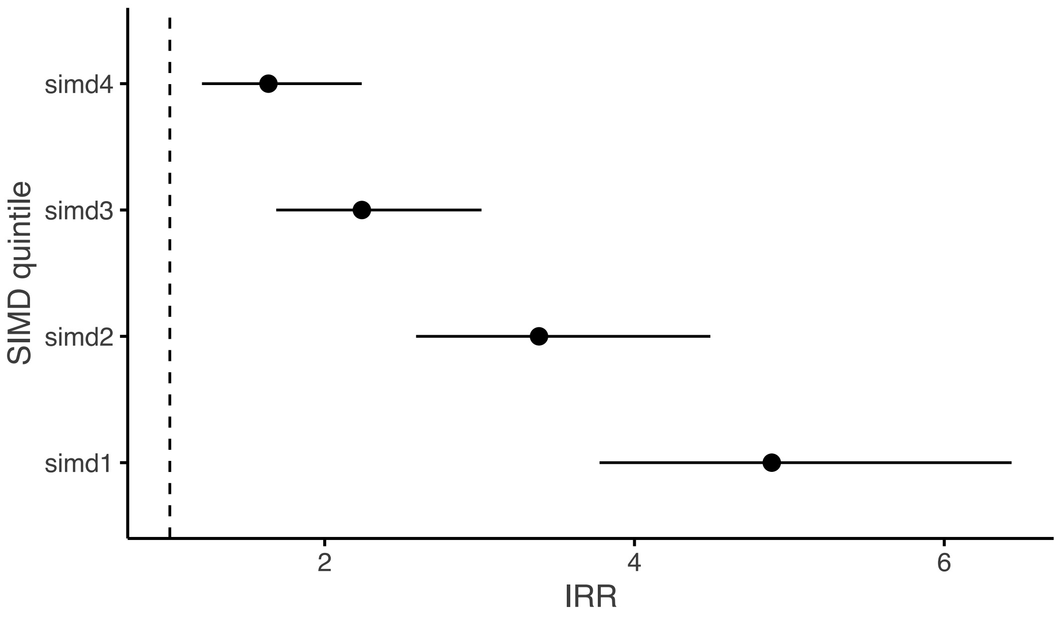

ESM figure 2. Incidence rate ratio for mortality by SIMD quintile.
The reference level is the least deprived quintile.

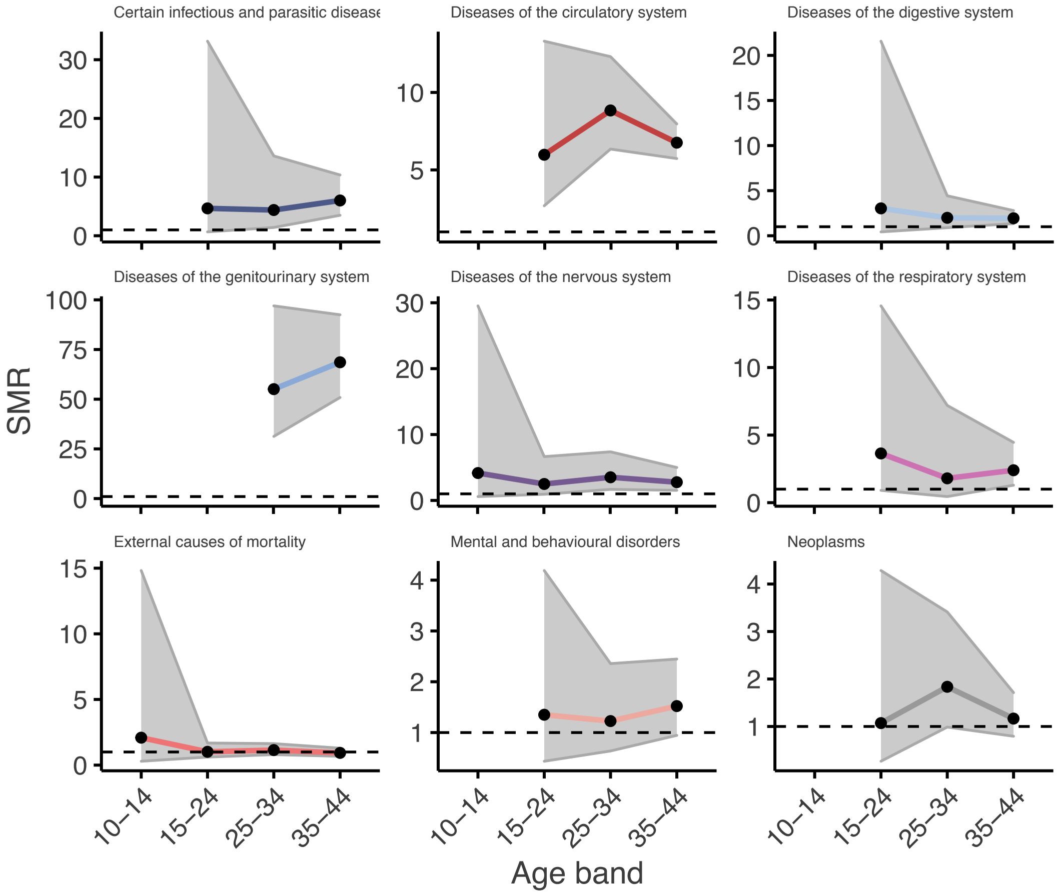

ESM figure 3. Standardised mortality ratio (SMR) by speciic cause and age band. For many combinations of specific cause and age band there are 0 deaths in the cohort and consequently no SMR can be calculated.
